# Monitoring the evolutionary aspect of the Gene Ontology to enhance predictability and usability

**DOI:** 10.1186/1471-2105-9-S3-S7

**Published:** 2008-04-11

**Authors:** Jong C Park, Tak-eun Kim, Jinah Park

**Affiliations:** 1 Computer Science Division, KAIST, 373-1 Guseong-dong, Yuseong-gu, Daejeon, 305-701, South Korea; 2 School of Engineering, Information & Communications University, Yuseong-gu, Daejeon, 305-732, South Korea

## Abstract

**Background:**

Much effort is currently made to develop the Gene Ontology (GO). Due to the dynamic nature of information it addresses, GO undergoes constant updates whose results are released at regular intervals as separate versions. Although there are a large number of computational tools to aid the development of GO, they are operating on a particular version of GO, making it difficult for GO curators to anticipate the full impact of particular changes along the time axis on a larger scale. We present a method for tapping into such an evolutionary aspect of GO, by making it possible to keep track of important temporal changes to any of the terms and relations of GO and by consequently making it possible to recognize associated trends.

**Results:**

We have developed visualization methods for viewing the changes between two different versions of GO by constructing a colour-coded layered graph. The graph shows both versions of GO with highlights to those GO terms that are added, removed and modified between the two versions. Focusing on a specific GO term or terms of interest over a period, we demonstrate the utility of our system that can be used to make useful hypotheses about the cause of the evolution and to provide new insights into more complex changes.

**Conclusions:**

GO undergoes fast evolutionary changes. A snapshot of GO, as presented by each version of GO alone, overlooks such evolutionary aspects, and consequently limits the utilities of GO. The method that highlights the differences of consecutive versions or two different versions of an evolving ontology with colour-coding enhances the utility of GO for users as well as for developers. To the best of our knowledge, this is the first proposal to visualize the evolutionary aspect of GO.

## Background

Much organized and sustained effort is currently made to develop the Gene Ontology (GO)[1]. The primary purpose of GO is to provide a uniform terminology in the form of a structured vocabulary to annotate gene products in genome databases, developed concurrently in the world, e.g., to reach a total of 968 in the NAR online Molecular Biology Database Collection as of November 2006 [2]. In addition to the rapidly growing amount of database information that the GO curators need to account for, they must also resolve such important issues as internal consistency [3,4] as well as external transparency [5]. Due to the dynamic nature of information it addresses, GO goes through constant updates whose results are released at monthly intervals as separate versions.

There are a large number of computational tools, such as AmiGO, DAGEdit, and GO-TermFinder, to aid the development process of GO [6], but such tools are inherently static, in the sense that they are operating only on a particular version of GO. It is thus up to the GO curators to keep a separate record of all the evolutionary changes that GO goes through, but it is hard to anticipate the full impact of particular changes along the time axis on the larger scale in this manner, leaving inconsistency inevitably behind. Proposals are also made in the literature to extend GO semi-automatically [7], by applying text mining techniques to the literature database such as MEDLINE, but they still fall short of establishing the level of confidence that the GO curators require. There is thus clearly a need for an intuitive method to consult the timeline of changes to GO, so that GO curators would be able to see the full impact of the proposed changes quickly and to identify the direction of future changes readily.

Such a service would be useful not only for GO curators, who can move back and forth along the different versions of GO to examine the possible room for improvement, but also for occasional users of GO, who may not have up-to-date information about a particular branch of molecular biology but are puzzled at some of the aspects that are in the middle of important changes. We present a method for tapping into such an evolutionary aspect of GO, by making it possible to keep track of important temporal changes to any of the nodes and relations of GO and by consequently making it possible to anticipate and overcome such changes. To the best of our knowledge, this is the first proposal to visualize the evolutionary aspect of GO.

## Methods

We developed graph construction and visualization methods to monitor the changes in GO over time. The system is a web-based application that is implemented by using GraphViz and Python. Currently, there are 58 GO versions (from April 2005 to September 2007) loaded into our system. Given two versions of GO, i.e., (GO_*t1*_, GO_*t2*_) where GO_*t1*_ temporally precedes GO_*t2*_, we compute the difference between two GOs and keep the GO terms with a tagging to distinguish how each term is changed: added, removed, or modified. This tagged information is used for colour-coding each node of the layered graph, which is constructed by merging some interested parts of the two versions of GO. A node in the graph refers to a GO term. The following is the node colour-coding convention we employed in our visualization.

1. Pink nodes indicate GO terms that have different concept names but use the same GO identifiers in the two versions of GO.

2. Red nodes indicate GO terms that appear in GO_*t1*_ (i.e., the older version) but that do not appear in GO_*t2*_ (i.e., the more recent version).

3. Blue nodes indicate GO terms that are newly added to GO_*t2*_ (i.e., the more recent version).

4. Gray nodes indicate GO terms that undergo no changes in both concept names and GO identifiers between the two versions of GO.

**Figure 1:**
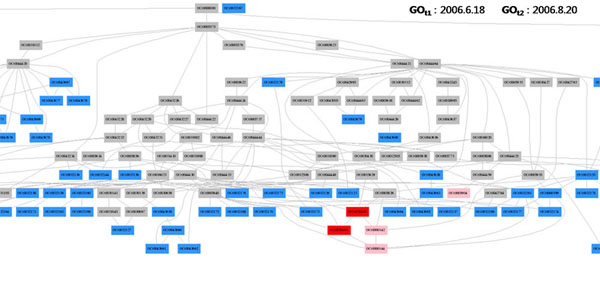
**GO Colour-Coding**. There are 2 red nodes (removed nodes), 3 pink nodes (modified nodes), and a number of blue nodes (added nodes) and gray nodes (unchanged nodes) computed from June 2006 GO version and August 2006 GO version.

In addition, we use the edge colour-coding so that the *isa* edges and *partof* edges are coloured blue and orange, respectively. Figure 1 shows 2 red nodes, 3 pink nodes and a number of blue nodes and gray nodes. For the purpose of clear exposition, the nodes in Figure 1 and in the following figures are designed to show only GO identifiers, but not the relation (or edge) type. We developed graph construction algorithms to merge only those subgraphs of interest from the whole GO.

**Figure 2:**
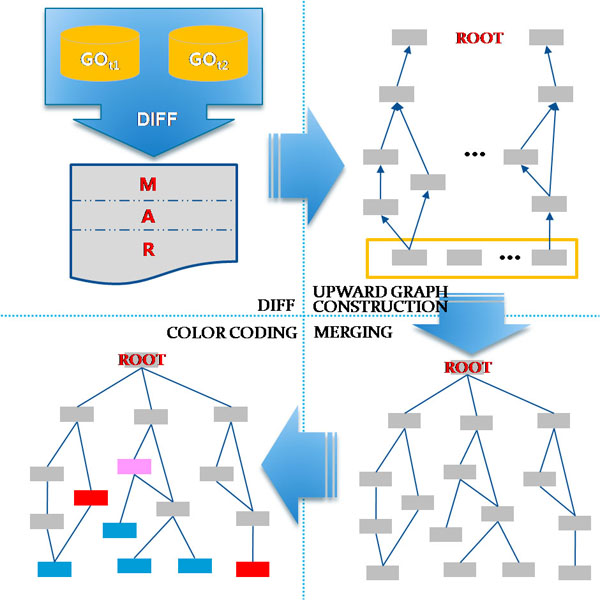
**Upward Graph Construction.** The lists M, A, R, each containing modified nodes, added nodes, and removed nodes, respectively, are computed from the two versions of GO, GO_t1_ and GO_t2_, by the procedure DIFF. Each element of the lists M, A, R is used as a leaf node for the upward traversal to the root node, constructing an individual subgraph. These subgraphs are subsequently merged together, and colour-coded.

### Upward/downward graph construction

The procedure UPWARD_GRAPH_CONSTRUCT takes two versions of GO, where GO_t1_ temporally precedes GO_t2_, and returns a colour-coded subgraph that contains all the nodes that are added (colour-coded blue), removed (colour-coded red), or modified (colour-coded pink). The nodes that are unchanged, colour-coded gray, are shown only when they participate in the graph construction for the leaf nodes to traverse upwards to reach the root node of GO. In Line 1 of the body of the procedure UPWARD_GRAPH_CONSTRUCT, the subprocedure DIFF takes the two versions of GO and returns the lists M, R, and A, which contain the modified nodes, removed nodes, and added nodes, respectively. Figure 2 illustrates the involved subprocesses graphically.

**PROCEDURE:** UPWARD_GRAPH_CONSTRUCT(GO_t1_, GO_t2_):

- ***CONSTRAINT:*** GO_*t1*_ temporally precedes GO_*t2*_

- ***INPUT:*** GO_t1_*// GO version 1*

GO_t2_*// GO version 2*

- ***OUTPUT:****colour-coded merged graph*

**1** M, R, A = DIFF(GO_t1_, GO_t2_)

**2** upward_subgraphs ← NULL

**3** for each GO_ID ∈ M ∪ A ∪ R

**4**  *queue* ← insert GO_ID

**5**  *edge* ← NULL

**6**  while *queue* is not empty

**7**   *current* ← delete *element* in *queue*

**8**   if *current* ∈ M ∪ R

**9**    isa_parents ← { ‘isa’ parent GO_IDs in GO_t1_ }

**10**    partof_parents ← { ‘part-of’ parent GO_IDs in GO_t1_ }

**11**   else if *current* ∈ M ∪ A

**12**    isa_parents ← { ‘isa’ parent GO_IDs in GO_t2_ }

**13**    part of_parents ← { ‘part-of’ parent GO_IDs in GO_t2_ }

**14**   for each pGO_ID in is a_parents

**15**    *queue* ← insert pGO_ID

**16**    *edge* ← *edge* ∪ (pGO_ID, current, ‘isa’)

**17**   for each pGO_ID in part of_parents

**18**    *queue* ← insert pGO_ID

**19**    *edge* ← *edge* ∪ (pGO_ID, current, ‘partof’)

**20**  upward_subgraphs ← upward_subgraphs ∪ *edge*

**21** G ← MERGE_SUBGRAPHS(upward_subgraphs)

**22** return COLOUR_CODING(G, M, R, A)

The procedures DIFF, MERGE_SUBGRAPHS, and COLOUR_CODING are shown in the Additional File 1.

**Figure 3:**
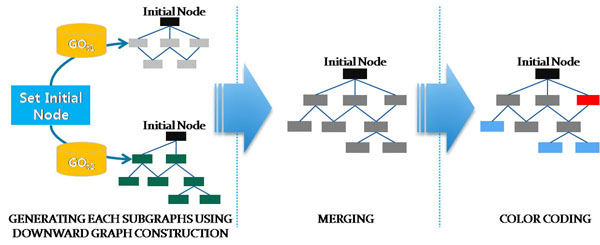
**Downward Graph Construction.** Given a particular GO term as an initial node, two subgraphs of the initial node as a root is constructed from two versions of GO. These two subgraphs are merged together and colour-coded subsequently.

The procedure DOWNWARD_GRAPH_CONSTRUCT is similarly defined, except that isa_parent and partof_parent should be replaced with isa_children and partof_children, respectively, and that it takes a specific GO term as an initial node. Figure 3 shows the process of constructing the initial subgraphs that are merged together and colour-coded subsequently.

**PROCEDURE:** DOWNWARD_GRAPH_CONSTRUCT(GO_t1_, GO_t2_, INITIAL_GO_ID)

- ***CONSTRAINT:*** GO_*t1*_ temporally precedes GO_*t2*_

- ***INPUT:*** GO_t1_, GO_t2_

- ***OUTPUT:****colour-coded merged graph*

**1** M, R, A = DIFF(GO_t1_, GO_t2_)

**2** downward_subgraphs ← NULL

**3*** queue* ← insert INITIAL_GO_ID

**4** for each GO_ID ∈ M ∪ A ∪ R

**5**  *queue* ← insert GO_ID

**6**  *edge* ← NULL

**7**  while *queue* is not empty

**8**   *current* ← delete *element* in *queue*

**9**   if *current* ∈ M ∪ R

**10**    isa_children ← { ‘isa’ children GO_IDs in GO_t1_ }

**11**    partof_children ← { ‘part-of’ children GO_IDs in GO_t1_ }

**12**   else if *current* ∈ M ∪ A

**13**    isa_children ← { ‘isa’ children GO_IDs in GO_t2_ }

**14**    partof_children ← { ‘part-of’ children GO_IDs in GO_t2_ }

**15**   for each cGO_ID in isa_children

**16**    *queue* ← insert cGO_ID

**17**    *edge* ← *edge* ∪ (cGO_ID, current, ‘isa’)

**18**   for each cGO_ID in partof_children

**19**    *queue* ← insert cGO_ID

**20**    *edge* ← *edge* ∪ (cGO_ID, current, ‘partof’)

**21**  downward_subgraphs ← downward_subgraphs ∪ *edge*

**22** G ← MERGE_SUBGRAPHS(downward_subgraphs)

**23** return COLOUR_CODING(G, M, R, A)

Note that the method to keep track of the changes between two versions of GO as described above can be naturally extended to the changes among multiple versions of GO.

## Results

By using visualization methods for viewing the changes between two different versions of GO through a layered graph and by colour-coding GO entries of addition, deletion and content modification in the two versions, we were able to infer the cause of the evolution as well as to gain new insights into more complex changes. We demonstrate the utility of our system with several examples.

### Making hypotheses

**Figure 4:**
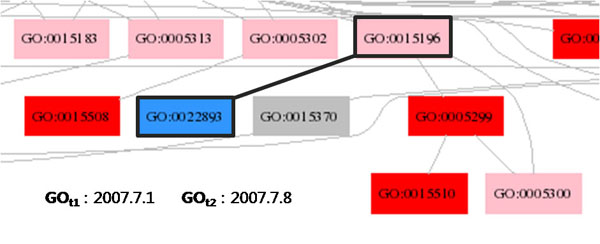
**Making Hypotheses I.** When a blue node has a pink node as its parent, it is likely that the pink node has a revised concept name to accommodate the addition of its child node.

**Figure 5:**
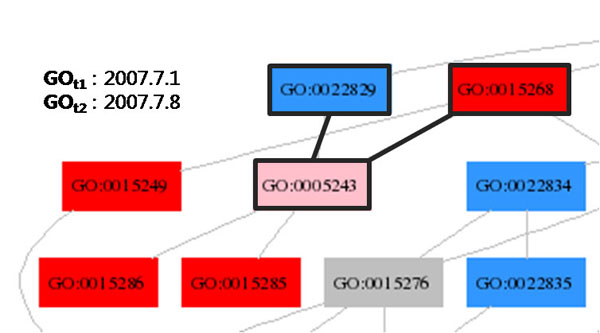
**Making Hypotheses II.** It is likely that the red node GO:0015268 is replaced with the blue node GO:0022829, given their contexts in the subgraph.

**Figure 6:**
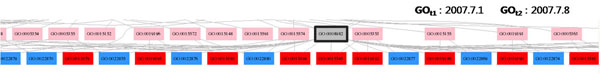
**Making Hypotheses III.** When the parent node has a big fan-out (i.e., a large number of children nodes) and there are similar numbers of red children nodes and blue children nodes, it is likely that each red node is replaced with a blue node.

We can make useful hypotheses by using the procedure UPWARD_GRAPH_CONSTRUCT and by focusing on coloured nodes that have certain structural properties with their neighbour nodes. These hypotheses can be checked manually by consulting the involved concept names, and adjusted subsequently for a more fine-tuned hypothesis. For the purposes of illustration, we use Figures 4,5 and 6 to explain possible hypotheses that one can make.

There are two possibilities for the reason that the node GO:0015196 in Figure 4 is modified (therefore it is coloured in pink). First, when a blue node has a pink node as its parent, it is likely that the parent node, i.e., the pink node, has a revised concept name to accommodate the addition of the blue node as its child node. The pink node GO:0015196, indeed, has its name changed from *L-tryptophan transporter activity* to *L-trptophan transmembrane transporter activity*. Note that the blue node GO:0022829 has the concept name *low-affinity tryptophan transmembrane transporter activity*. Second, it is also likely that the node GO:0015196 is coloured pink because of its changed relation with the node GO:00005299, which is removed, and with the node GO:0005300, which is directly linked to the node GO:0015196 as its child. In this particular case, however, it is more likely that the changed content of the GO term is due to the blue node, not due to the red node.

When a red node and a blue node share the same parent and the same child, it is likely that the red node is replaced with the blue node. In Figure 5, the nodes GO:0015268 and GO:0022829 have concept names *Alpha-type channel activity* and *Wide pore channel activity*, respectively.

When the parent node has a big fan-out (i.e., a large number of children nodes), as in Figure 6, we can think of two cases. First, there may be similar numbers of red children nodes and blue children nodes. In this case, it is likely that each red node has been replaced with a blue node. The fan-out of the node GO:0008982 in this figure is 26, and there are equal numbers (13 each) of red nodes and blue nodes under it. As we see in Table 1, the concept names of the red nodes are systematically changed to those of the blue nodes. Second, there may be a large number of pink nodes, along with some gray nodes. In this case, we can also expect that the pink nodes are the result of systematic changes in the concept names. In both cases, when there is a parent node with a big fan-out, it is possible that it is the result of the agreement among the GO curators [5]. Even if the fan-out is small, when there are equal numbers of red nodes and blue nodes, the chance is quite high that the red nodes are replaced into the blue nodes.

**Table 1:** Mapping Old GOIDs to New GOIDs (ref: Figure 6).

**OLD GOID**	**OLD CONCEPT**	**NEW GOID**	**NEW CONCEPT**
GO:0015579	glucose permease activity	GO:0022855	protein-N(PI)-phosphohistidine-glucose phosphotransferase system transporter activity
GO:0019195	galactosamine porter activity	GO:0022876	protein-N(PI)-phosphohistidine-galactosamine phosphotransferase system transporter activity
GO:0015580	N-acetylglucosamine permease activity	GO:0022880	protein-N(PI)-phosphohistidine-N-acetylglucosamine phosphotransferase system transporter activity
GO:0015584	trehalose permease activity	GO:0022879	protein-N(PI)-phosphohistidine-trehalose phosphotransferase system transporter activity
GO:0015585	fructose permease activity	GO:0022877	protein-N(PI)-phosphohistidine-fructose phosphotransferase system transporter activity
GO:0019192	fructose porter activity		
GO:0019189	lactose permease activity	GO:0022869	protein-N(PI)-phosphohistidine-lactose phosphotransferase system transporter activity
GO:0019190	cellobiose permease activity	GO:0022874	protein-N(PI)-phosphohistidine-cellobiose phosphotransferase system transporter activity
GO:0015581	maltose porter activity	GO:0022873	protein-N(PI)-phosphohistidine-maltose phosphotransferase system transporter activity
GO:0015582	beta-glucoside permease activity	GO:0022882	protein-N(PI)-phosphohistidine-beta-glucoside phosphotransferase system transporter activity
GO:0015586	mannitol permease activity	GO:0022872	protein-N(PI)-phosphohistidine-mannitol phosphotransferase system transmembrane transporter activity
GO:0015587	sorbitol permease activity	GO:0022856	protein-N(PI)-phosphohistidine-sorbitol phosphotransferase system transporter activity
GO:0015588	galactitol permease activity	GO:0022875	protein-N(PI)-phosphohistidine-galactitol phosphotransferase system transmembrane transporter activity
		GO:0022870	protein-N(PI)-phosphohistidine-mannose phosphotransferase system transporter activity

### Monitoring the evolutionary behaviour

The following descriptions on Figures 7 through 10 (for GO:0031399), Figures 11 through 14 (for GO:0048622), and Figure 15 through 17 (for GO:0016331) show how to use the procedure DOWNWARD_GRAPH_CONSTRUCT to gain insights into the evolutionary nature of a subgraph under a particular GO node. (See Additional File 2 for Figures 7 through 17.)

As we illustrate in Figures 7 through 10 (see Additional File 2), the node GO:0031399 *regulation of protein modification* did not exist until the 2005.4.18 version of GO, when it was introduced along with the other 5 nodes. Since GO is represented in a DAG form, the two nodes with the same parent node are not necessarily on the same level in the GO hierarchy. Nonetheless, it appears that such a level can be assessed in the GO hierarchy with respect to the nodes in the subgraph with the node GO:0031399 as its root node. In particular, the node GO:0031396 *regulation of protein ubiquitination* has two sibling nodes GO:0030401 *positive regulation of protein modification* and GO:0030397 *negative regulation of protein modification*, that is, a pair of nodes with the opposing qualifiers *positive* and *negative*. In the case of the concept *ubiquitination*, which has to traverse down one level to see the opposing qualifiers, the levels of detail for the terms do not appear overall quite adequate, and we can expect that they will undergo a subsequent refinement process. Similarly, the newly introduced node GO:0033234 has the concept name *positive regulation of protein sumoylation*, but the other newly introduced node GO:0033235 has the concept name *11-beta-hydroxysteroid dehydrogenase activity*, and it is thus likely that one of the nodes will undergo a name change in the future, as they do not follow the naming convention which involves the qualifiers *positive* and *negative*. As another example, we use the node GO:0048622 *reproductive sporulation* to show its evolutionary behaviour over time.

Figure 11 (see Additional File 2) shows that the corresponding GO terms are all very well understood and organized at the time of introduction. However, the existing node GO:0048315 becomes a child node of the node GO:0030437, thereby increasing its fan-out to 4, but leaving all its children nodes as leaf nodes (Figure 12; Additional File 2). In this case, it is highly likely that they will undergo a subsequent refinement process, as it is the usual tendency to put all the related but unrefined nodes as the children nodes of a certain parent node until the classification method matures. This prediction is borne out in Figure 13 (see Additional File 2), where the graph is restructured with additions and updates of the children nodes of the node GO:0030437. The fan-out of the node GO:0048622 becomes 1 again as the node GO:0048236 is coloured red (Figure 14; Additional File 2). Since its fan-out is now 1, it is again likely that the node GO:0048622 would be merged into another node, such as into its child node GO:0030437, or another node is added to it as its child node, so as to increase its fan-out to 2 or more.

Finally, we examine cases that require more reasoning to monitor the evolutionary behaviour. First, consider the subgraph under the node GO:0016331 in the 2006.1.22 version of GO (Figure 15; Additional File 2). Note that there is no intervening node between the nodes GO:0001841 and GO:0001842. Figure 16 (see Additional File 2) shows that in the 2006.2.19 version of GO, a new node, GO:0014020, along with its subgraph (except for the node GO:0001842, which is already included in the graph), is added to the graph, though the two nodes GO:0001841 and GO:0001842 retain their relationship to each other. Table 5 (see Additional File 2) shows the individual concept names of the involved nodes.

Note that *neural fold formation* (GO:0001842) is considered a refined concept of both *primary neural tube formation* (GO:0014020) and *neural tube formation* (GO:0001841). At this point, we may predict that this arrangement for *neural fold formation* (GO:0001842) is not natural, and that one of the two links, most likely the latter with *neural tube formation* should be disconnected. Note also that the four nodes, GO:0014024, GO:0014022, GO:0014023, and GO:0014025, are introduced at the same time as the parent node GO:0014020.

For about 7 months afterwards, however, the subgraph under the node GO:0014020 did not undergo any changes, until the 2006.8.20 version of GO is introduced, where quite a large number of nodes are added to the subgraph of the node GO:0001840, with concept names changes (e.g., GO:0001840) to accommodate the new addition (Figure 17; Additional File 2). Note that our prediction earlier is borne out in the new version of GO, where the connection between the two nodes GO:0001841 and GO:0001842 is no longer maintained.

We have examined several cases to illustrate the process of making hypotheses and validating them with subsequent changes. We are also examining how often such cases appear, but this requires a semi-automated method to search for similar cases. We have used cases in the past to make validation possible as well, but the method is surely applicable to cases in the future, where one can make a prediction as to where changes of a particular type are inevitable or imminent. The use of our colour-coded graphs allows the users to zoom in and out at ease and rapidly focus on a GO term or GO terms of interest to monitor the evolutionary behaviour.

The software as described in this paper has a web interface, accessible at .

## Discussion and conclusion

The need to keep track of the changes to GO has also been recognized by Yeh et al. [4], where two versions of GO are compared by the use of a collection of tools called PROMPT, which generates a list of differences and similarities between the two input ontologies. In addition, the OntoViz tab generates a graphical representation of concepts in GO, facilitating the process of inspecting such changes. However, the focus of the work by Yeh et al. is on ensuring the consistency of the involved ontologies over possible changes, and not on providing a means to tap into the evolutionary process of GO itself.

Ensuring the consistency of an ontology is certainly an important aspect for the GO development, as there are numerous researchers constantly contributing materials to GO, possibly leading to conflicts of various nature. Nonetheless, we find that it is equally important to help developers and users of GO to move around different versions of GO along the time axis so as to recognize emerging (or overlooked) trends quickly and to determine a course of action to exploit the trends. Such emerging trends cannot be detected if we are to look at only a static snapshot of GO. Our contribution in this paper through the implemented software is to make available a means for monitoring changes of GO over time, so that both developers and users of GO may understand the full impact of such changes on the larger scale.

There are, however, a number of remaining issues that must be overcome. First, the system may generate a collection of red nodes when it compares two versions of GO, or GO_t1_ and GO_t2_ where GO_t1_ temporally precedes GO_t2_, but such a collection of red nodes can not be interpreted to have been just removed from the version of GO_t1_, especially when there is a big temporal gap between the two versions of GO. To see precisely when such nodes have been removed from a particular version of GO, we need to locate two versions, GO_t_i_ and GO_t_i+1_ where GO_t1_ temporally precedes GO_t_i_, GO_t_i_ temporally precedes GO_t_i+1_, there is no published version of GO between GO_t_i_ and GO_t_i+1_, and GO_t_i+1_ temporally precedes GO_t2_, such that the nodes are gray in GO_t_i_ but red in GO_t_i+1_. It is quite unlikely though that the nodes are all gray in GO_t_i_ and all red in GO_t_i+1_, but more likely that they show individual differences from one another in the points of removal. In this regard, one can not make a decisive conclusion when one sees red nodes, unless the comparison is made between two consecutive versions of GO.

Second, we have shown a number of scenarios that demonstrate the utility of the proposed visualization method, but these scenarios are the result of a somewhat unfocused search over random pairs of the available versions of GO, mostly guided by the intuition of a system developer. It would obviously help much if the search process can be automated for the detection of “meaningful” scenarios. Such an automated search process would be particularly useful to help suggest a possible direction for improvement or refinement over the present version of GO to GO curators. For instance, we have used a hypothesis with respect to Figure 6 such that similar numbers of red and blue nodes in a tree with a big fan-out ratio suggest a particular activity. It is, however, quite possible that there may be disagreements among the GO curators on the utility of such a hypothesis. It would hence be quite tricky to use it as a universal condition for the automated search process, unless such disagreements can be overcome. It would also help much if we can get a more global perspective over the changes of GO, so that we can get a sense of the frequency or the proportion of the meaningful cases in designing the automated search process.

While limitations of this kind must be overcome, we believe that the present system that highlights the differences of two versions of an evolving ontology such as GO can be effectively used for the development of the ontology in the future, as well as for the identification of meaningful scenarios in the past.

## Competing interests

The authors declare that they have no competing interests.

## Authors' contributions

JCP conceived and directed this work. JCP also drafted the initial version of the paper. TK implemented all the modules of the presented system and prepared all the tables and figures. JP provided advice and feedback to the visualization side of the work. All authors contributed during the whole length of the project. All authors read and approved the final manuscript.

## Supplementary Material

Additional File 1 APPENDIX A: Pseudo code for sub-procedures
This file describes pseudo code for sub-procedures. Click here for file

Additional File 2 Additional file – Figures 7-17
This file contains Figures 7 to 17 and Tables 2 to 5, which illustrate DOWNWARD_GRAPH_CONSTRUCT to monitor the evolutionary behavior of a subgraph under a particular GO node. Click here for file
