# Supplementary material for: Monitoring the evolutionary aspect of the Gene Ontology to enhance predictability and usability
Source: BMC Bioinformatics. 2008 Apr 11;9(Suppl 3):S7. doi: 10.1186/1471-2105-9-S3-S7 (PMC2349298; doi:10.1186/1471-2105-9-S3-S7)
Supplement: Additional File 1 — APPENDIX A: Pseudo code for sub-procedures This file describes pseudo code for sub-procedures. [file 1471-2105-9-S3-S7-S1.doc]

**APPENDIX A: Pseudo code for sub-procedures**

**1. Sub-procedure for comparing differences of two versions of Gene Ontology**

This sub-procedure takes two Gene Ontology hierarchy graphs as input, compares their differences in GO IDs and concept (or node) names, and returns the GO IDs that show changes of concept names between the two versions of GO. In particular, the sub-procedure returns three sets of GO IDs, or M, R, and A. The set M includes GO IDs that are both present in the two versions of GO, but that show changed concept names. The set R includes those that used to be in GO_t1_ but are not present in GO_t2_. The set A includes those that are newly added in GO_t2_. GO_t1_ temporally precedes GO_t2_.

| **PROCEDURE:** DIFF(GO_t1_, GO_t2_):   - ***INPUT:*** GO_t1_, GO_t2_ - ***OUTPUT:*** *M // modified nodes*   *R // removed nodes*  *A // added nodes*  **1** S1 ← { id: id ∈ GO_ID in GO_t1_ }  **2** S2 ← { id: id ∈ GO_ID in GO_t2_ }  **3** M ← { m: m ∈ S1 ∩ S2 and ConceptName_in_GO_t1_(m) ≠ ConceptName_in_GO_t2_(m) }  **4** R ← { r: r ∈ S1 – S2 }  **5** A ← { a: a ∈ S2 – S1 }  **6** return M, R, A |
| --- |

**2. Sub-procedure for merging subgraphs**

This sub-procedure takes a set of subgraphs as input and returns the merged graph. Each subgraph is defined as a set of edges, and the merged graph is constructed by connecting those edges that share the same Gene Ontology IDs (cf. Line 5). Line 4 works to eliminate redundant edges and to avoid multiple links between two nodes.

| **PROCEDURE:** MERGE_SUBGRAPHS(S)   - ***INPUT:*** *S // set of sub-graphs* - ***OUTPUT:*** *merged graph*   **1** *graph* ← NULL  **2** for each *subgraph* ∈ S  **3** for each *edge* ∈ *subgraph*  **4** if *edge* not in *graph*  **5** *graph* ← *graph* ∪ *edge*  **6** return *graph* |
| --- |

**3. Sub-procedure for colouring nodes of subgraphs**

This sub-procedure uses the results of the DIFF procedure over two versions of GO and returns the coloured concept (or node) names. If the concept names are changed, removed, or added, the corresponding nodes are coloured pink, red, or blue, respectively. The nodes with no changes are coloured gray.

| **PROCEDURE:** COLOUR_CODING(G, M, R, A)   - ***INPUT:*** *G // graph*   *M // modified nodes*  *R // removed nodes*  *A // added nodes*   - ***OUTPUT:*** *colour-coded graph*   **1** for each *node* in G  **2** if *node* ∈ M: colour *node* in pink.  **3** else if *node* ∈ R: colour *node* in red.  **4** else if *node* ∈ A: colour *node* in blue.  **5** else: colour *node* in gray.  **6** return colour-coded graph G’ |
| --- |
