# Supplementary material for: Monitoring the evolutionary aspect of the Gene Ontology to enhance predictability and usability
Source: BMC Bioinformatics. 2008 Apr 11;9(Suppl 3):S7. doi: 10.1186/1471-2105-9-S3-S7 (PMC2349298; doi:10.1186/1471-2105-9-S3-S7)
Supplement: Additional File 2 — Additional file – Figures 7-17 This file contains Figures 7 to 17 and Tables 2 to 5, which illustrate DOWNWARD_GRAPH_CONSTRUCT to monitor the evolutionary behavior of a subgraph under a particular GO node. [file 1471-2105-9-S3-S7-S2.pdf]

Additional file - Figures 7-17 and Tables 2 to 5

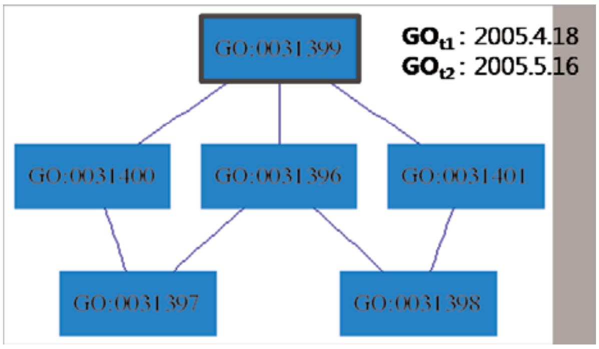

Figure 7. Monitoring the Evolutionary Behaviour I (1/4). All the nodes are newly introduced during 2005.4.18 ~ 2005.5.16. The subgraph is constructed by DOWNWARD_GRAPH_CONSTRUCT on the two versions of GO and the node GO:0031399 regulation of protein modification.

Table 2 - Nodes under GO:31399 during 2005.5.16 ~ 2005.11.20 *(ref: Figure 7).*

GOID	CONCEPT
GO:0031399	regulation of protein modification
GO:0031400	negative regulation of protein modification
GO:0031396	regulation of protein ubiquitination
GO:0031401	positive regulation of protein modification
GO:0031397	negative regulation of protein ubiquitination
GO:0031398	positive regulation of protein ubiquitination

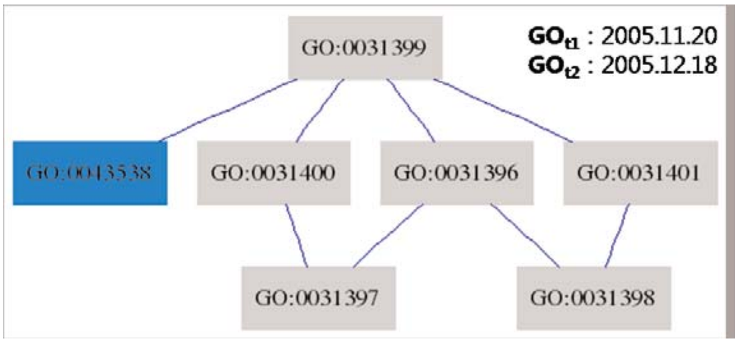

Figure 8. Monitoring the Evolutionary Behaviour I (2/4). During 2005.11.20 ~ 2005.12.18, a new node, GO:0043538 regulation of actin phosphorylation, is introduced as a child to the node GO:0031399.

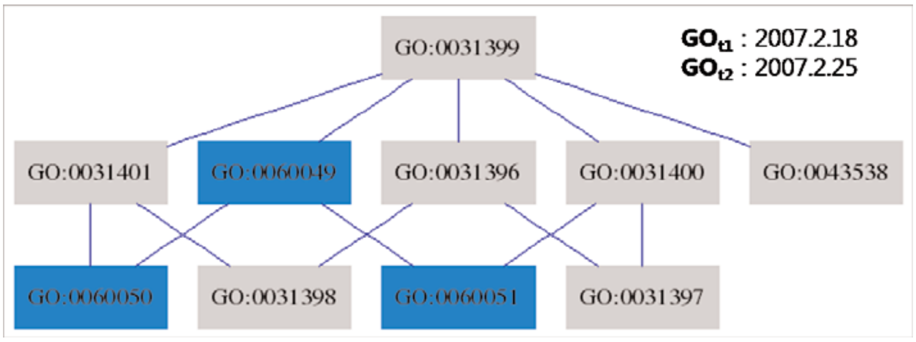

Figure 9. Monitoring the Evolutionary Behaviour I (3/4). During 2007.2.18 ~ 2007.2.25, three more nodes, GO:0060049, GO:0060051, and GO:0060050, are newly introduced.

Table 3. New nodes under GO:31399 during 2007.2.25 ~ 2007.5.27 *(ref: Figure 9)*.

GOID	CONCEPT
GO:0060049	regulation of protein amino acid glycosylation
GO:0060050	positive regulation of protein amino acid glycosylation
GO:0060051	negative regulation of protein amino acid glycosylation

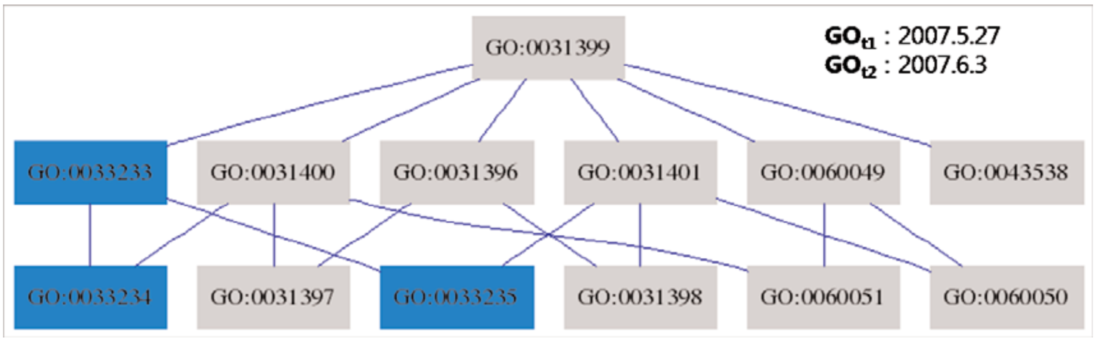

Figure 10. Monitoring the Evolutionary Behaviour I (4/4). Finally, during 2007.5.27 ~ 2007.6.3, three more nodes are added.

Table 4. During 2007.6.3 ~ 2007.9.16, three more nodes are added *(ref: Figure 10)*.

GOID	CONCEPT
GO:0033233	regulation of protein sumoylation
GO:0033234	positive regulation of protein sumoylation
GO:0033235	11-beta-hydroxysteroid dehydrogenase (NAD+) activity

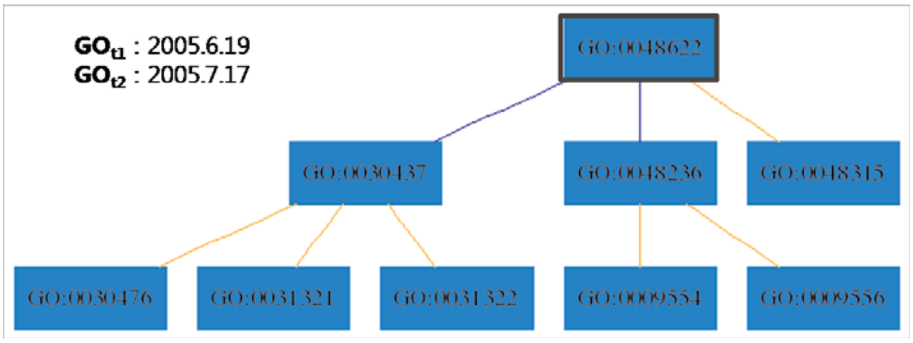

Figure 11. Monitoring the Evolutionary Behaviour II (1/4). All the 9 nodes are newly introduced during 2005.6.19 ~ 2005.7.17, including the node GO:0048622.

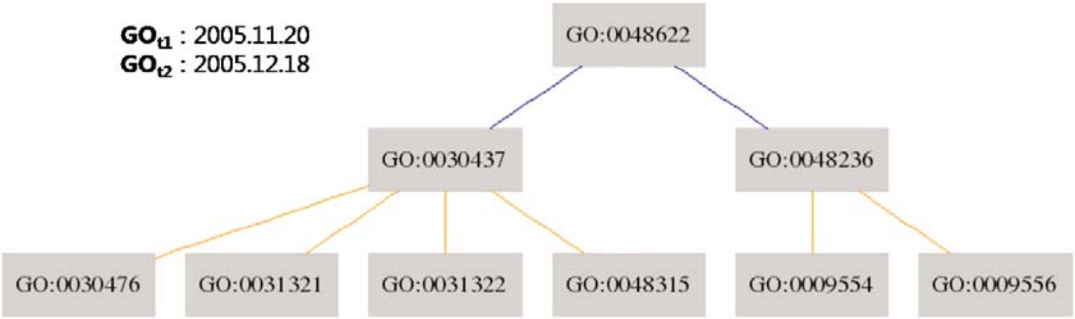

Figure 12. Monitoring the Evolutionary Behaviour II (2/4). The node GO:0048236 is rearranged during 2005.11.20 ~ 2005.12.18, but does not show any difference in colour.

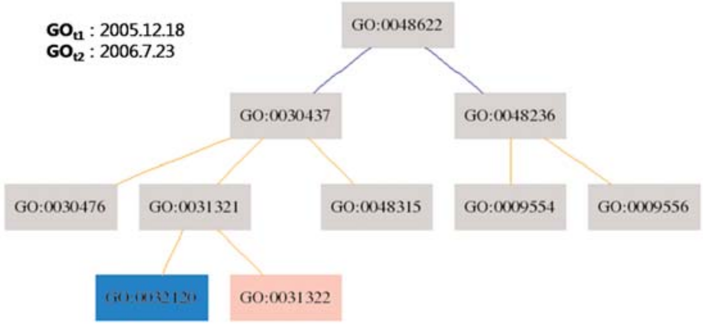

Figure 13. Monitoring the Evolutionary Behaviour II (3/4). The changes to the subgraph under the node GO:0048622 during 2005.12.18 ~ 2006.7.23 show that it is likely that the pink node GO:0031322 changed its concept name to accommodate its addition to the node GO:0031321 as its child, though it is still also a child of the node GO:0030437.

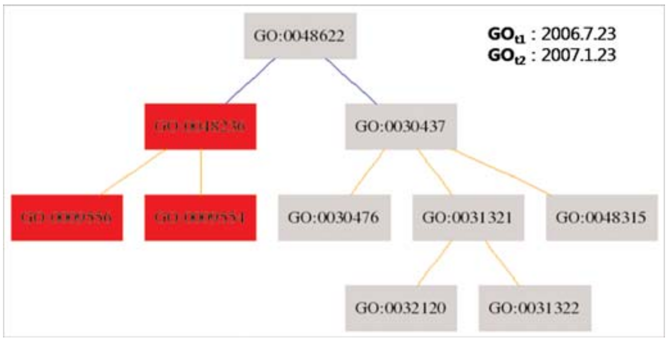

Figure 14. Monitoring the Evolutionary Behaviour II (4/4). During 2006.7.23 ~ 2007.1.23, the node GO:0048236 and its own subgraph (a total of three nodes) are removed entirely from the larger subgraph under the node GO:0048622.

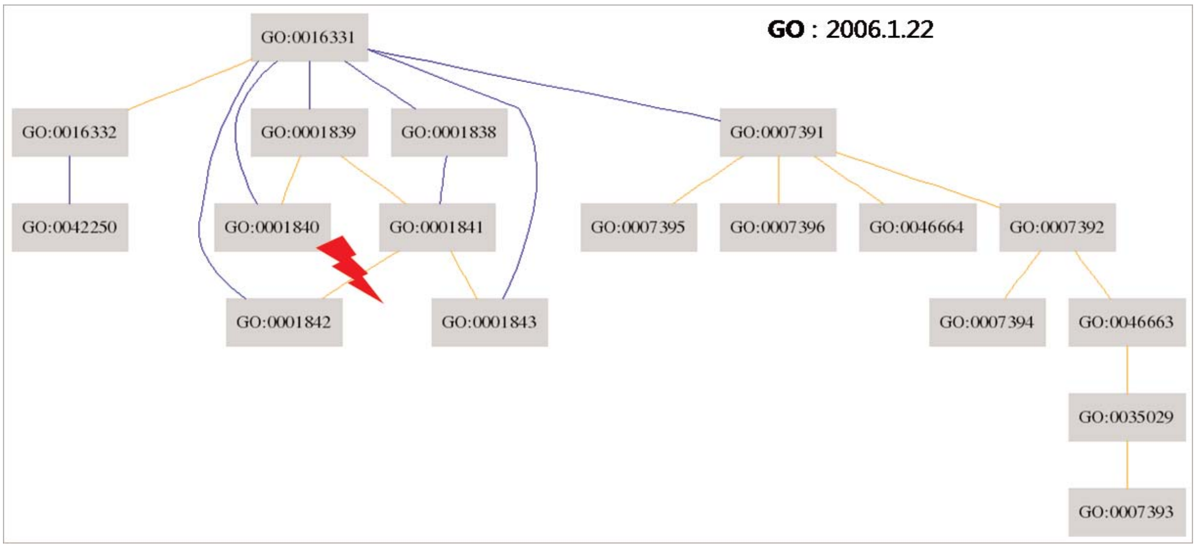

Figure 15. Monitoring the Evolutionary Behaviour III (1/3). There is no intervening node between the nodes GO:0001841 and GO:0001842 in the 2006.1.22 version of GO.

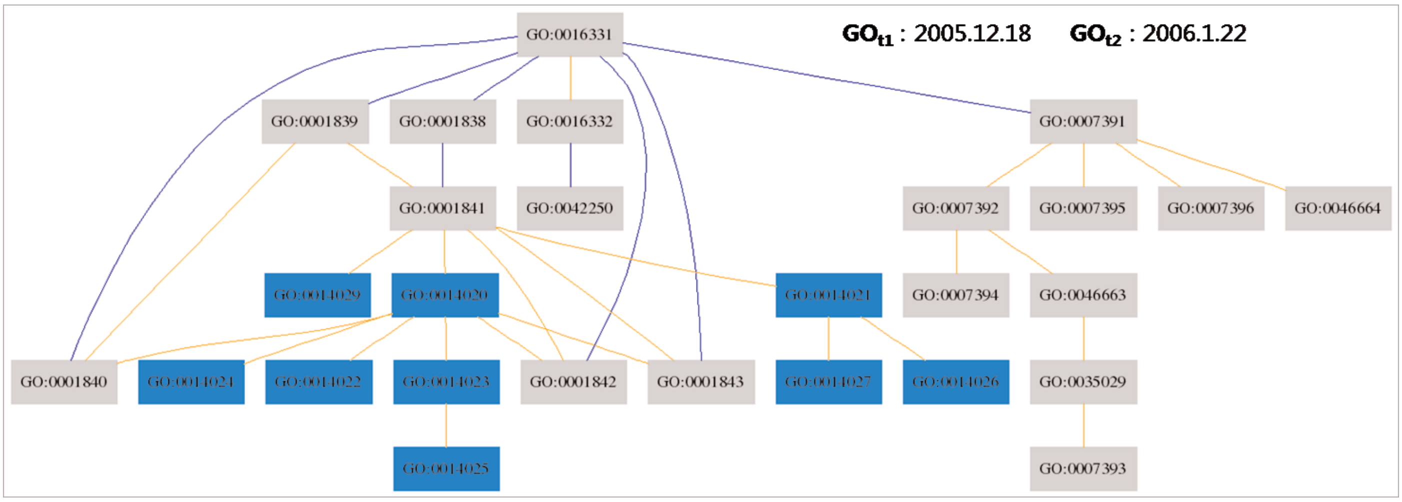

Figure 16. Monitoring the Evolutionary Behaviour III (2/3). The two nodes GO:0001841 and GO:0001842 retain their relationship, but a new node, GO:0014020, along with its subgraph (except the node GO:0001842, which is already in the graph), is added to the existing graph.

Table 5. Monitoring the Evolutionary Behaviour *(ref: Figure 16).*

GOID	CONCEPT
GO:0016331	morphogenesis of embryonic epithelium
GO:0001839	neural plate morphogenesis
GO:0001838	embryonic epithelial tube formation
GO:0001841	neural tube formation
GO:0014020	primary neural tube formation
GO:0001842	neural fold formation
GO:0014029	neural crest formation
GO:0014022	neural plate shaping
GO:0014023	neural rod formation
GO:0014024	neural rod cavitation
GO:0001843	neural tube closure

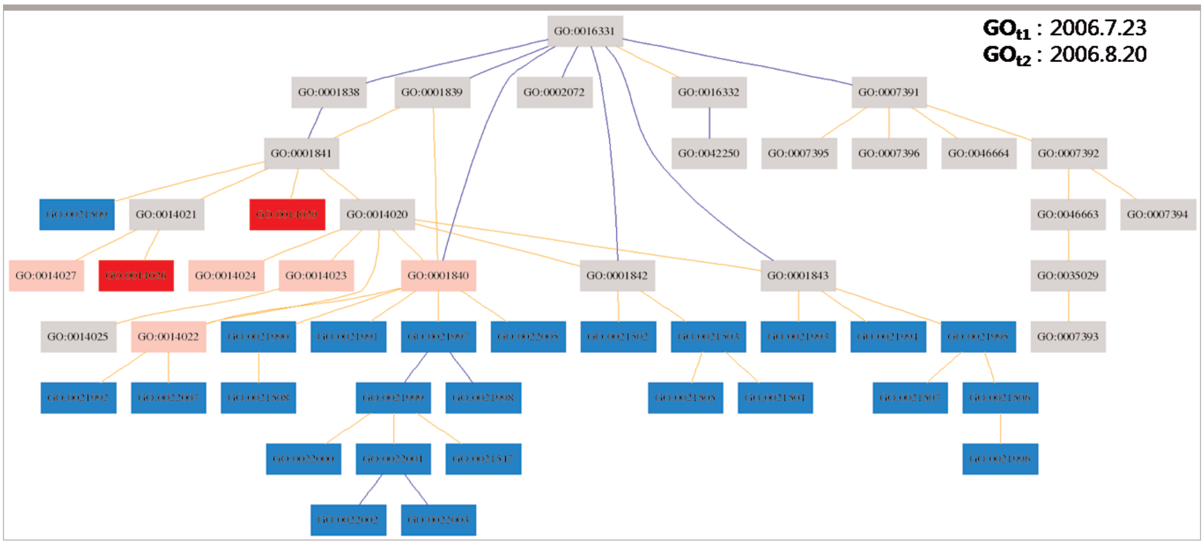

Figure 17. Monitoring the Evolutionary Behaviour III (3/3). A dormant subgraph is suddenly expanded massively.
